# Simulation-based Comparison of British and Australian Advanced Life Support Guidelines

**DOI:** 10.5811/westjem.59836

**Published:** 2023-10-09

**Authors:** Fawaz Altuwaijri

**Affiliations:** King Saud University, College of Medicine, Department of Emergency Medicine, Riyadh, Saudi Arabia

**Keywords:** cardiopulmonary resuscitation, Australian Resuscitation Council, Resuscitation Council United Kingdom, Adult Life Support

## Abstract

**Introduction:**

Cardiac arrest is a major health concern that has been linked to poor disease outcomes. Cardiopulmonary resuscitation (CPR) is a critical protocol for restoring spontaneous circulation. The guidelines used by medical staff differ across different countries. A comparison of these guidelines can help in designing more efficient Advanced Life Support (ALS) protocols. The goal in this study was to compare the guidelines for interruption of compression during CPR (hands-off time) for ALS protocols provided by Australian and United Kingdom (UK) resuscitation councils.

**Methods:**

The author designed a simulation-based study using a mannequin and a defibrillator, and then recruited six participants. Three participants were certified ALS practitioners who followed UK guidelines, and three were certified ALS practitioners who followed Australian guidelines. Each participant received a random task assignment for each scenario, as a team leader, performer of cardiopulmonary resuscitation, or assistant. The team leader and the chest compressor were unaware of the shockability of each case’s rhythm. Eight minutes total were spent on 10 CPR trials, each lasting four cycles. A video of the simulation was recorded for automated timekeeping. An independent sample *t*-test was used to compare the amount of hands-off time (seconds) throughout each cycle between two procedures. For purposes of calculating statistical significance, a 0.05 *P*-value was employed.

**Results:**

The mean duration of second cycle hands-off time (seconds) in the UK ALS protocol was statistically significantly longer than the Australian ALS (*t* = −2.100; *P* = 0.05). For shockable rhythms, the hands-off time of the UK ALS protocol was significantly longer than Australian ALS protocol, as reflected in the second cycle (*t* = −0.621; *P* < 0.001), third cycle (*t* = −8.083; *P* < 0.001), and fourth cycle (*t* = −5.814; p < 0.001), while the difference in the first cycle between groups was not statistically significant. (*t* = −0.258; *P* = 0.803).

**Conclusion:**

This simulation-based study demonstrated that the UK ALS guidelines led to an increased duration of hands-off time during the second cycle. The hands-off time in the shockable rhythms was also higher during the second, third, and fourth cycles in the UK ALS protocol compared to the Australian ALS protocol. These points must be focused on in future revisions of the UK ALS guidelines. For better results, it is critical to limit hands-off time between chest compression cycles.

Population Health Research Capsule What do we already know about this issue?

*Cardiopulmonary resuscitation (CPR) is critical in restoring spontaneous circulation in cardiac arrest, but national protocols vary.*

What was the research question?

*The goal was to compare the hands-off time recommended by the Australian and UK resuscitation councils and identify more efficient advanced life-saving protocols.*

What was the major finding of the study?

*The mean duration of the hands-off time in shockable rhythms in the UK ALS guidelines was significantly longer than in the Australian ALS guidelines (t = −2.100; P = 0.05).*

How does this study improve population health?

*By minimizing hands-off time between chest compression cycles, the quality of chest compressions can be enhanced, leading to improved outcomes in cardiac arrest cases.*

## INTRODUCTION

Cardiac arrest is a serious public health concern that has been linked to a high incidence of mortality.^1^ Both out-of-hospital and in-hospital cardiac arrests are associated with poor disease outcomes.^2^ ^,^ ^3^ Chest compression qualities, including proper depth and pace, appropriate chest recoil and, critically, minimal interruptions, are necessary to increase the survival rates of cardiac arrest patients. When treating a person experiencing shockable cardiac arrest, interruptions typically refer to the time required to monitor their rhythm, pulse, intravenous cannulation, intubation, and administration of a shock if necessary.^4^ The cardiac output produced by effective chest compressions is roughly 30% of the average value. It has been demonstrated that stopping chest compressions reduces coronary perfusion pressures, cardiac output, and brain perfusion pressures.^5^

High-quality cardiopulmonary resuscitation (CPR) is an established practice crucial for the restoration of spontaneous circulation and effective outcomes in cardiac arrests. CPR can deliver blood to the major organs at an adequate level of coronary perfusion pressure.^6^ More recent guidelines have focused on improving survival rates by improving CPR quality.^7^ Edelson et al found that performing high-quality CPR, defibrillation as soon as possible and reducing hands-off time—defined as the total number of breaks between chest compressions during each cycle of CPR—improved survival rates.^8^

Recent guidelines advise a maximum hands-off time per cycle of ≈5 seconds.^9^ Prior research has shown that a shorter hands-off period enhances the likelihood of survival.^10^ More recent studies emphasize the need to minimize interruptions between chest compressions cycles to improve the chest compression quality and attain better outcomes.^4^ ^,^ ^11^ Duration of peri-shock pause—defined as the time consumed before and after delivering the shock—was found to be inversely related to outcomes in animal studies.^12^

Advanced Life Support (ALS) guidelines from the Resuscitation Council United Kingdom (RCUK) state that the rescuer should continue CPR until the defibrillator is retrieved and pads applied. The shocks must be given with minimal interruptions to minimize the pre- and post-shock pauses.^13^ In contrast, the adult ALS guidelines of the Australian Resuscitation Council (ARC) recommend charging the shock immediately while performing chest compressions, so that the defibrillator is charged and ready upon rhythm check if deemed necessary.^14^

The effectiveness of charging the manual defibrillator during chest compressions before pausing to monitor the rhythm has been assessed in several human and mannequin trials.^15^ ^,^ ^16^ Depending on whether a shockable heart rhythm is discovered, the defibrillator may be armed or disarmed. Pre-charging technique minimises pauses and hands-off time overall.^15^ ^,^ ^17^ ^,^ ^18^ The difference in pre-charging protocols can affect the hands-off time, which can determine the harm during chest compressions.^15^ The variation in these protocols warrants a comparison to develop consensus guidelines. In this study, the author for the first time compared the hands-off time duration in a cardiac arrest between the ALS protocols provided by British and Australian resuscitation guidelines.

## METHODOLOGY

The author conducted a simulation-based study in a medical simulation facility, where the experiments were run using a Resusci Anne mannequin (Laerdal Medical Corporation, Stavanger, Norway) and a LIFEPAK 20 (Physio-Control Inc., Redmond, WA) defibrillator. Six participants were enrolled from a tertiary-care hospital in Riyadh, Saudi Arabia. Participants’ cohort allocation was based on ALS certification, either by the RCUK or the ARC. They were allocated to one of two groups, with three participants in each group. The first group followed RCUK protocols, and the second group followed the protocols established by the Australian Resuscitation Council (ARC). Each participant, whether a team leader, CPR performer, or defibrillator assistant, was randomly assigned a specific task for every scenario.

Commands and rhythm checks fell under the purview of the team leader. The assistant oversaw administering medication, defibrillation, and ventilation. To eliminate bias, the defibrillator assistant retained 10 cards with various rhythms (pulseless electrical activity, pulseless ventricular tachycardia, asystole, and ventricular fibrillation), and participants were asked to choose one card for each situation. A brief patient history was given at the beginning of each case to mimic genuine cases. Ten CPR attempts lasting four cycles and a total of eight minutes were made. A video of the simulation was recorded for automated timekeeping.

## STATISTICAL ANALYSIS

The author used mean and standard deviation for the presentation of descriptive statistics. Hands-off time (seconds) in each cycle between Australian and UK ALS protocols was contrasted employing an independent sample *t*-test. For purposes of calculating statistical significance, a 0.05 *P*–value was employed. SPSS version 26 was used for all data analysis (IBM Corporation, Armonk, NY).

## RESULTS

Table 1 compares hands-off time in seconds between Australian and UK ALS protocols. We found that the mean duration of the second cycle hands-off time (seconds) following the RCUK protocol was statistically significantly longer than Australian ALS protocol (*t* = −2.100; *P* = 0.05), while the difference in the hands-off times of the first cycle, third cycle, and fourth cycle were not substantially different in Australian and UK ALS (*P* > 0.05). Table 1 presents the comparison of hands-off time in seconds between Australian and UK ALS protocols. We found that the mean duration of the second cycle hands-off time (seconds) in UK ALS was statistically significantly longer than Australian ALS (*t* = −2.100; *P* = 0.05), while the difference in the hands-off times of the first, third, and fourth cycles were not significantly different in Australian and UK ALS (*P* > 0.05).

**Table 1.** Descriptive statistics of the hands-off time between Australian and United Kingdom Advanced Life Support protocols.

Cycle level	Hands-off time in seconds		*t*-test	*P*-value^§^
	Australia ALS Mean ± SD	UK ALS Mean ± SD		
First cycle	5.20 ± 1.23	5.40 ± 0.97	−0.405	0.691
Second cycle	4.80 ± 1.39	6.10 ± 1.37	−2.100	0.050^**^
Third cycle	4.80 ± 1.48	5.80 ± 1.48	−1.515	0.147
Fourth cycle	4.80 ± 1.55	6.20 ± 1.81	−1.856	0.080

^§^ *P*-value calculations are based on an independent sample *t*-test.

^**^ Significance threshold at *P* ≤ 0.05.

*ALS*, Advanced Life Support; *UK*, United Kingdom.

Table 2 compares hands-off tine in shockable rhythms between Australian and UK ALS. It can be observed that the hands-off time (seconds) of the UK ALS protocol was statistically significantly longer than the Australian ALS, which was reflected in the second cycle (*t* = −0.621; *P* < 0.001), third cycle (*t* = −8.083; *P* < 0.001), and fourth cycle (*t* = −5.814; *P* < 0.001) while the difference in the first cycle was not statistically significant between the groups (*t* = −0.258; *P* = 0.803).

**Table 2.** Comparison of hands-off time in shockable rhythms between Australian and United Kingdom Advanced Life Support protocols.

Cycle level	Hands-off time in seconds		*t*-test	*P*-value^§^
	Australia ALS Mean ± SD	UK ALS Mean ± SD		
First cycle	5.20 ± 1.64	5.40 ± 0.55	−0.258	0.803
Second cycle	3.80 ± 0.45	6.60 ± 0.89	−6.261	**<0.001** ^**^
Third cycle	3.60 ± 0.55	6.40 ± 0.55	−8.083	**<0.001** ^**^
Fourth cycle	3.60 ± 0.55	6.20 ± 0.84	−5.814	**<0.001** ^**^

^§^ *P*-value calculations are based on an independent sample *t*-test.

^**^ Significance threshold at *P* ≤ 0.05.

*ALS*, Advanced Life Support; *UK*, United Kingdom.

Table 3 shows the comparative analysis of the hands-off time in the non-shockable rhythms between the Australian and British ALS. The mean ± SD hands-off times are lower for the first and fourth cycles in Australian ALS as compared to the UK ALS (5.20 ± 0.84 vs 5.40 ± 1.34 and 6.00 ± 1.22 vs 6.20 ± 2.59, respectively) and higher in the second and third cycles (5.80 ± 1.30 vs 5.60 ± 1.67 and 6.00 ± 1.00 vs 5.20 ± 1.92, respectively). However, none of these differences were statistically significant (*P*-value > 0.05).

**Table 3.** Comparison of hands-off time in non-shockable rhythms between Australian and United Kingdom Advanced Life Support protocols.

Cycle level	Hands-off time in seconds		*t*-test	*P*-value^§^
	Australia ALS Mean ± SD	UK ALS Mean ± SD		
First cycle	5.20 ± 0.84	5.40 ± 1.34	−0.283	0.784
Second cycle	5.80 ± 1.30	5.60 ± 1.67	0.211	0.838
Third cycle	6.00 ± 1.00	5.20 ± 1.92	0.825	0.433
Fourth cycle	6.00 ± 1.22	6.20 ± 2.59	−0.156	0.880

^§^ *P*-value calculations are based on an independent sample *t*-test. In comparing time off-chest in non-shockable rhythms between Australian and UK ALS, it was found that all cycle levels were not significantly different in both Australian and UK ALS (*P* > 0.05).

*ALS*, Advanced Life Support; *UK*, United Kingdom.

## DISCUSSION

To the best of our knowledge, this is the first simulation-based study to compare hands-off time between the ALS guidelines provided by the UK and Australian resuscitation councils. This study demonstrated that the mean duration of second cycle hands-off time (seconds) in the UK ALS was statistically significantly longer than in the Australian ALS protocol (*t* = −2.100; *P* = 0.050. However, the difference in hands-off times of the first, third, and fourth cycles were not significantly different when comparing both Australian and UK ALS protocols (*P* > 0.05). The hands-off time is an important contributor to the overall success of CPR and can have life-saving importance.^2^ This finding clearly suggests that the Australian guidelines are more efficient at reducing the time between cycles, as interruptions between chest compressions can reduce the overall quality of CPR.^19^

Cardiac arrest is usually classified into shockable vs non-shockable. This classification is based on the electrocardiograph rhythm. The non-shockable rhythms are asystole and pulseless electrical activity (PEA). The two shockable rhythms are ventricular fibrillation and pulseless ventricular tachycardia. Administering CPR or a defibrillator to shock the heart within a few minutes may be used to reverse cardiac arrest in patients with shockable rhythms. Comparing hands-off time in shockable rhythms showed that these times were longer in the UK than in the Australian guidelines. The correlation was found to be statistically significant (*P* < 0.001). These more prolonged interruptions were evident in the second (*P* < 0.001), third (*P* < 0.001), and fourth (*P* < 0.001) cycles. However, the difference in the first cycle was not statistically significant when comparing both groups (*P* = 0.803). The difference was not found to be statistically significant for non-shockable rhythms (*P* > 0.05). These findings suggest that the Australian ALS guidelines address the time-off chest more closely by defibrillator pre-charging approach. To increase the effectiveness of the UK ALS protocol, the time-off chest may need to be addressed.^20^

## LIMITATIONS

Our study has certain limitations, including its single-center setting and simulation-based design, which hampered the measurement of mortality and morbidity. Another limitation was the unavailability of means to directly measure coronary perfusion pressures while performing CPR.

## CONCLUSION

The guidelines for ALS are based on the systemic analysis of the published evidence and grading of overall confidence in evidence and the strength of recommendations. A consensus is then developed through the participation of global stakeholders and clinicians. Analysis of these guidelines from time to time can lead to improvement in these protocols and enhance their overall efficiency. We found that the hands-off times in shockable rhythms were higher during the second, third, and fourth cycles in the UK ALS protocol compared to the Australian protocol. These points must be focused on in future revisions of UK ALS guidelines. Chest compression interruptions should be kept to a minimum for improved outcomes.
